# Cell Elasticity Is Regulated by the Tropomyosin Isoform Composition of the Actin Cytoskeleton

**DOI:** 10.1371/journal.pone.0126214

**Published:** 2015-05-15

**Authors:** Iman Jalilian, Celine Heu, Hong Cheng, Hannah Freittag, Melissa Desouza, Justine R. Stehn, Nicole S. Bryce, Renee M. Whan, Edna C. Hardeman, Thomas Fath, Galina Schevzov, Peter W. Gunning

**Affiliations:** 1 Oncology Research Unit, School of Medical Sciences, UNSW Australia, Sydney, NSW 2052, Australia; 2 Neurodegeneration and Repair Unit, School of Medical Sciences, UNSW Australia, Sydney NSW 2052, Australia; 3 Biomedical Imaging facility, UNSW Australia, Sydney, NSW 2052, Australia; 4 Neuromuscular and Regenerative Medicine Unit, School of Medical Sciences, UNSW Australia, Sydney, NSW 2052, Australia; LAAS-CNRS, FRANCE

## Abstract

The actin cytoskeleton is the primary polymer system within cells responsible for regulating cellular stiffness. While various actin binding proteins regulate the organization and dynamics of the actin cytoskeleton, the proteins responsible for regulating the mechanical properties of cells are still not fully understood. In the present study, we have addressed the significance of the actin associated protein, tropomyosin (Tpm), in influencing the mechanical properties of cells. Tpms belong to a multi-gene family that form a co-polymer with actin filaments and differentially regulate actin filament stability, function and organization. Tpm isoform expression is highly regulated and together with the ability to sort to specific intracellular sites, result in the generation of distinct Tpm isoform-containing actin filament populations. Nanomechanical measurements conducted with an Atomic Force Microscope using indentation in Peak Force Tapping in indentation/ramping mode, demonstrated that Tpm impacts on cell stiffness and the observed effect occurred in a Tpm isoform-specific manner. Quantitative analysis of the cellular filamentous actin (F-actin) pool conducted both biochemically and with the use of a linear detection algorithm to evaluate actin structures revealed that an altered F-actin pool does not absolutely predict changes in cell stiffness. Inhibition of non-muscle myosin II revealed that intracellular tension generated by myosin II is required for the observed increase in cell stiffness. Lastly, we show that the observed increase in cell stiffness is partially recapitulated in vivo as detected in epididymal fat pads isolated from a Tpm3.1 transgenic mouse line. Together these data are consistent with a role for Tpm in regulating cell stiffness via the generation of specific populations of Tpm isoform-containing actin filaments.

## Introduction

The structural and architectural properties of cells are regulated by the cytoskeleton, which is comprised of three polymer systems, actin filaments, microtubules and intermediate filaments. To date, numerous studies conducted on different cell model systems, using various experimental techniques, have convincingly demonstrated that cellular cortical stiffness is largely determined by the actin cytoskeleton [1–8]. Microtubules have minimal impact on cell stiffness and intermediate filaments contribute little to cortical stiffness, but play a major role in regulating intracellular mechanics [9–12].

The actin cytoskeleton is regulated by a plethora of actin binding proteins and specific signalling pathways [13], leading to the generation of distinct actin filament networks displaying unique dynamics and organizational properties [14,15]. For example, the mesh-like structure of the cortical actin filaments underlying the plasma membrane is different to the branched filaments found in lamellipodia, the parallel filaments within the filopodia and the different subtypes of stress fibers (dorsal, ventral, transverse arcs) that form within the cell [16,17]. A complex array of over 15 different types of actin filament structures have been identified in metazoans [14] that can change in both spatial and temporal intracellular distribution in response to physical and environmental stimuli.

Previous work demonstrating a relationship between the actin cytoskeleton and the mechanical properties of the cell have employed the use of actin filament disrupting drugs (cytochalasin D) or have microscopically visualised the actin cytoskeleton with the filamentous actin detecting compound, phalloidin [2,18]. These techniques, however, are unable to discriminate between the distinct actin filament populations known to exist within cells. Reconstituted F-actin networks *in vitro*, decorated with different actin binding proteins or synthetic cross-linker molecules generating distinct F-actin networks, have been shown to have dramatically different mechanical responses [19,20].

The actin filament associated protein tropomyosin (Tpm) is known to play a critical role in the generation of functionally distinct actin filaments [21–27]. Multiple Tpm isoforms are generated from 4 mammalian genes, giving rise to over 40 Tpm isoforms [24]. The distinct spatial and temporal subcellular distribution of these isoforms together with the ability to regulate the association of actin binding proteins to the filament, are shown to define the roles of actin filaments in an isoform-specific manner [21–27]. We thus hypothesise that distinct actin filament populations defined by different Tpm isoforms can differentially impact on the mechanical properties of cells.

In the current study we analysed the elastic properties of cells both *in vitro* and *in vivo* with altered Tpm isoform expression. We show for the first time that populations of actin filaments defined by their association with specific Tpm isoforms, differentially contribute to cell stiffness and impact on the mechanical properties of the cell. Furthermore, we show that the role of the actin cytoskeleton in the control of cortical cell stiffness is far more complex than a simple relationship between polymerised actin and stiffness.

## Materials and Methods

### Cell Culture

Rat neuroblastoma cells, B35 [28,29] stably transfected with different Tpm isoforms were maintained in DMEM supplemented with 2 mM L-glutamine, 10% fetal bovine serum (FBS) (Invitrogen, Life Technologies, Melbourne, VIC, Australia) and 0.6% Geneticin (Invitrogen) at 37°C in a humidified atmosphere of 5% CO_2_. B35 cells stably overexpressing the following tropomyosin isoforms have previously been described; Tpm1.10, Tpm3.1 and Tpm1.12 [21,30]; Tpm1.11 and Tpm4.2 [31]; and Tpm1.7 [32]. For the present study, the Tpm2.1 overexpressing B35 clones were generated by inserting the rat Tpm2.1 cDNA between the 5’ SalI and 3’ BamHI sites of the phβAPr3 (sig-) vector [33]. Transfection was performed using Fugene according to manufacturer's instructions (Roche, Sydney, NSW, Australia) and transfected clones selected using 1% Geneticin (Invitrogen).

### Atomic Force Microscopy on cells *in vitro*

Cells (5 x10^4^) were seeded on 40 mm glass bottom cell culture dishes (Willco Wells BV, Amsterdam, Netherlands), previously coated with poly-D-lysine (0.1 mg/ml) (Sigma Aldrich, Sydney, Australia). Cells were cultured for 24 hrs and the media was changed to remove any debris and/or dead cells prior to AFM measurements. The Bioscope Catalyst (Bruker, Germany) mounted on a Leica DMI 3000 B inverted optical microscope equipped with a stage heater set at 37°C within a TMC vibration isolation table (Technical Manufacturing Corporation, MA, USA), was used for both cell imaging and indentation. Before each experiment, the AFM system was calibrated as previously described [34]. In brief, the deflection sensitivity of the probe was measured in fluid by engaging the probe on an uncoated glass substrate, withdrawal of the probe and a thermal tune sweep is then used to determine the spring constant. Finally, the tip radius is estimated via a tip-check sample.

A V-shaped cantilever with a pyramidal Scan Assist fluid tip probe (Veeco, Camarillo, CA, USA) with a nominal tip radius of 20 nm, a spring constant of 0.7 N/m and a resonance frequency of 120–180k Hz was selected and mounted on the fluid holder of the AFM scanner. For determination of the elastic modulus, indentation in Peak Force Tapping mode [35] with an average loading force of 600 pN and a scan rate of 0.1 μm/s was performed at a region just above the nucleus giving an indentation depth of 500 nm. The indentation depth was chosen following the analyses of force versus indentation curves generated with various tip loading forces (0.1 nN to 2 nN) [36]. Cells with similar morphologies, not in contact with each other, were measured in culture media and measurements per dish of cells were conducted for no longer than 2 hrs to ensure that optimal cell viability was maintained throughout the measurements. An average of 15–20 force curves were recorded out of a maximum of 20 indentations per cell. To minimize the effect of successive indentations on membrane deformation, a 3 secs rest between each indentation point was applied. The Young’s modulus was extracted from force curves using the Sneddon fit [37] (Eq 1) and Nanoscope Analysis software (version 1.40, Bruker). Representative force curves fitted with Sneddon’s equation are shown (S1 Fig).

### Atomic Force Microscopy on epididymal fat

All animal experiments were performed in accordance with UNSW Australia Animal Care and Ethics Committee approval (11.132B) and the National Health and Medical Research Council guidelines. The overexpressing Tpm3.1 [F-Tg(*ACTB*-*Tpm3*.Tm5NM1)52Pgun] TG mouse line has been previously described [21,38]. The epididymis fat pads were dissected from adult male mice, 6 per group. The specimens, usually harvested from several animals at once, were stored in PBS (2.6 mM NaH_2_PO_4_, 3 mM Na_2_HPO_4_, 155 mM NaCl, 0.01% NaN_3_ (w/v), pH 7.0) supplemented with a protease inhibitor cocktail (Boehringer-Mannheim, Mannheim, Germany) at 4°C and processed for AFM measurements within 72 hrs of harvest. A 1% agarose (Bioline Australia, NSW, Australia) gel was prepared in PBS and a layer of agarose, approximately 0.2 mm thick, was poured onto a 40 mm glass bottom cell culture dish (Willco Wells BV). Prior to the gel setting completely, the tissues were immobilized within the agarose. The AFM system was calibrated as described for the cell experiments and a silicon nitride triangle cantilever with a 4.5 μm spherical colloidal probe (Novascan, Ames, IA, USA) with a spring constant of 0.06 N/m was selected. Indentation in Peak Force Tapping mode with an average loading force of 200 pN was performed on 5–10 different areas per tissue (30 ramp minimum per area) and 10–50 indentation points per area were conducted. The Young’s modulus values were calculated from force curves based on the Hertz spherical indentation model (Eq 2).

$$F=\frac{2}{\pi }\text{tan}\;\alpha \;\frac{E}{\left(1-\nu^{2}\right)}\delta^{2}$$ Equation 1

$$F=\frac{4}{3}\frac{E}{\left(1-\;\nu^{2}\right)}\sqrt{\delta^{3/2}\text{R}}$$ Equation 2

F = force, E = Young’s modulus, *v* = Poisson’s ratio and is related to the compressibility of the material (*v* = 0.5 in this study), R = radius of the indenter and δ = indentation depth and α = tip’s half angle.

### siRNA knockdown of Tpm isoforms

Transient knockdown of the exogenously expressed human Tpm3.1 and Tpm2.1 were performed with pre-annealed 3′ Alexa Fluor 488–labelled oligonucleotide duplexes. The target siRNA sequence for hTpm3.1 used was 5´-AAAAGCTGGAAGAAGCTGAAA-3´ and 5´-AAGCACATCGCTGAGGATTCA-3 for Tpm2.1 [32,39] and a scramble control siRNA (GE Dharmacon). Transfections were conducted using Lipofectamine 3000 (Life Technologies) reagent in OPTI-MEM I (Life Technologies) reduced serum medium. For harvesting total cellular protein and proceed with western blotting, 5 x 10^5^ cells per well of a 6-well plate was used. To proceed with AFM measurements cells were seeded as described above.

### Inhibition of non-muscle myosin II with blebbistatin

Exposure of cells to blebbistatin was performed by seeding cells onto 40 mm glass bottom Petri dishes (Willco Wells BV). Cells were treated for 30 min with blebbistatin (Merck Millipore, Billerica, MA, USA) dissolved in DMSO (Sigma Aldrich), final concentration 50 μM.

### Visualization of F-actin, image acquisition and analysis

Cells were seeded at 4 × 10^3^ cells/well, 8 wells per clone, in a 96-well plate (Cell Carrier-96 Black Optically Clear Bottom plates, PerkinElmer) and cultured for 24 hrs. The fixation and immunostaining of cells was performed as previously described [40] with minor modifications. In brief, cells were fixed in 4% (w/v) paraformaldehyde/phosphate-buffered saline (PBS) for 30 min and washed twice with PBS. Permeabilization and staining was performed in a one-step procedure using ATTO-488 Phalloidin 1/1000 (ATTO-Tec, Siegen, Germany), 0.1% Triton X-100 and DAPI at 1/10000 (Invitrogen, Life Technologies) in PBS and incubated for 60 min at room temperature in the dark. Cells were washed twice with PBS with the final wash left on the plate during imaging. The Opera High Content Screening System (PerkinElmer Life Sciences, CA, USA) equipped with a Nipkow spinning disc microscope and 20X Air LUCPLFLN NA = 0.45 objective was used to acquire images with the following settings: laser power of 7440 μW, 480 msec camera exposure, excitation at 488 nm to detect phalloidin; camera exposure of 40msec and excitation at 365 nm for DAPI. Images from eight randomly chosen microscopic fields/well were taken. A linear feature detection algorithm [40] was employed to quantitatively evaluate the organization of the actin cytoskeleton. Total actin filament bundle length per cell was measured from 3 independent experiments.

### SDS-PAGE, western blotting and quantitation

Cells or tissues were washed in PBS and protein extracted in radioimmunoprecipitation assay buffer (RIPA) (20 mM Tris pH 7.4, 150 mM sodium chloride, 1% Nonidet P-40, 0.5% sodium deoxycholate, 1 mM EDTA, 0.1% SDS) supplemented with a Complete Mini Protease Inhibitor EDTA free tablet (Roche) and a PhosSTOP phosphatase inhibitor cocktail tablet (Roche). Short pulses with a sonicator were used to solubilize both cells and tissues. Protein concentration was estimated using the Direct Detect infrared (IR)-based quantification, according to manufacturer's instructions (Merck Millipore). SDS-PAGE (12.5%) and Western blotting was performed as previously described [41]. Blots were probed with the following antibodies: mouse monoclonal γ/9d (1:500) detecting Tpm3.1 and Tpm3.2; rabbit polyclonal δ/9d (1:500) detecting Tpm4.2; rabbit polyclonal α/9c (1:500) detecting Tpm1.10 and Tpm1.12; sheep polyclonal α/9b (1:500) detecting Tpm1.11; mouse monoclonal Tm311 (1:500) (Sigma Aldrich), detecting Tpm1.10, Tpm2.1, Tpm1.6 and Tpm1.7 (all Tpm antibodies are described in [41]); mouse monoclonal α-tubulin (1:1000) (clone DM 1A, Sigma Aldrich); mouse monoclonal C4 actin (1:1000) [42]; mouse monoclonal vimentin (1:1000) (Sigma Aldrich) and mouse monoclonal Glyceraldehyde-3-phosphate Dehydrogenase (GAPDH) (1:1000) (Sigma Aldrich). Protein loading was also estimated following the staining of the blots with Ponceau stain [31]. Secondary antibodies used were: anti-rabbit, anti-sheep and anti-mouse Ig-conjugated horseradish peroxidise (HRP) (GE Healthcare, Sydney, NSW, Australia), and donkey/anti-rabbit/HRP antibodies (Jackson ImmunoResearch laboratories, Suffolk, UK). Blots were developed with the Western Lighting chemiluminescence reagent (PerkinElmer Life Sciences) and exposed to Fuji x-ray film (FUJIFILM Australia). Blots were quantified using ImageJ software and each lane expressed relative to GAPDH or Ponceau stain blot.

### Quantitation of Tpm and actin expression

To evaluate the expression levels of Tpm isoforms, known concentrations of His-tagged recombinant Tpm expression constructs were compared to cellular lysates. The generation of these constructs and isolation of recombinant Tpm protein has been previously described [41]. Serial dilutions of Tpm recombinant proteins (0.005 to 0.1 μg) together with 10 μg total protein lysates, isolated from four separate experiments, from the different Tpm-overexpressing B35 clones, were separated on a 12.5% SDS-PAGE gel. Gels were subsequently processed for western blotting and probed with the different Tpm antibodies. Densitometry was performed as described above and the amount of an individual Tpm in a cell was calculated (μg of Tpm protein expressed per μg of total cellular protein). To evaluate the expression of actin, recombinant skeletal muscle actin (Jomar Bioscience, SA, Australia) was used and the ng of actin per μg of total cellular protein calculated.

### G-actin/F-actin assay

The ratio of globular to filamentous actin was measured using an *in vitro* assay kit (Cytoskeleton Inc., Denver, CO, USA) as per manufacturer’s instructions. In brief, cells were cultured to 90% confluence in 100 mm washed with PBS and lysed with the F-actin stabilization and lysis buffer supplemented with 1 mM ATP and 1% protease inhibitor cocktail. Following an incubation period of 15 min at 37°C, samples were centrifuged at 2000rpm for 5 min. The supernatant was centrifuged at 134,000*g*, 37°C for 1 hr in an ultracentrifuge (CS150NX Tabletop Micro Ultracentrifuge, Hitachi Koki Co. Ltd., Japan) to partition the G-actin (supernatant) from the F-actin (pellet) fractions. The pellet was resuspended in 8 M urea (Cytoskeleton Inc.) incubated on ice for 1 hr and vortexed every 15 min to dissociate F-actin. The ratio of F-actin to G-actin was determined using Western blotting by probing with the actin antibody, followed by densitometry.

### Statistical analysis

Statistical analysis was performed using the nonparametric Mann-Whitney test with two tails at the 95% confidence interval (GraphPad Prism 5.0; GraphPad, La Jolla, CA, USA). The results are presented as standard error of the mean (SEM) and number of experiments denoted as *n*. Values of *P*< 0.05 were considered statistically significant. Elastic modulus data are visualized as box and whiskers plots/scatter dots with the median (horizontal line in the box), the borders of the boxes representing the 25% and 75% percentiles and the whiskers displaying outliers within 1.5 interquartile range of the lower and upper quartile, respectively. The data were analysed by Kruskal-Wallis-Anova nonparametric test followed by Dunnett's multiple comparison test.

## Results

### Tpm isoforms differentially impact the elastic modulus of a cell

**Fig 1:**
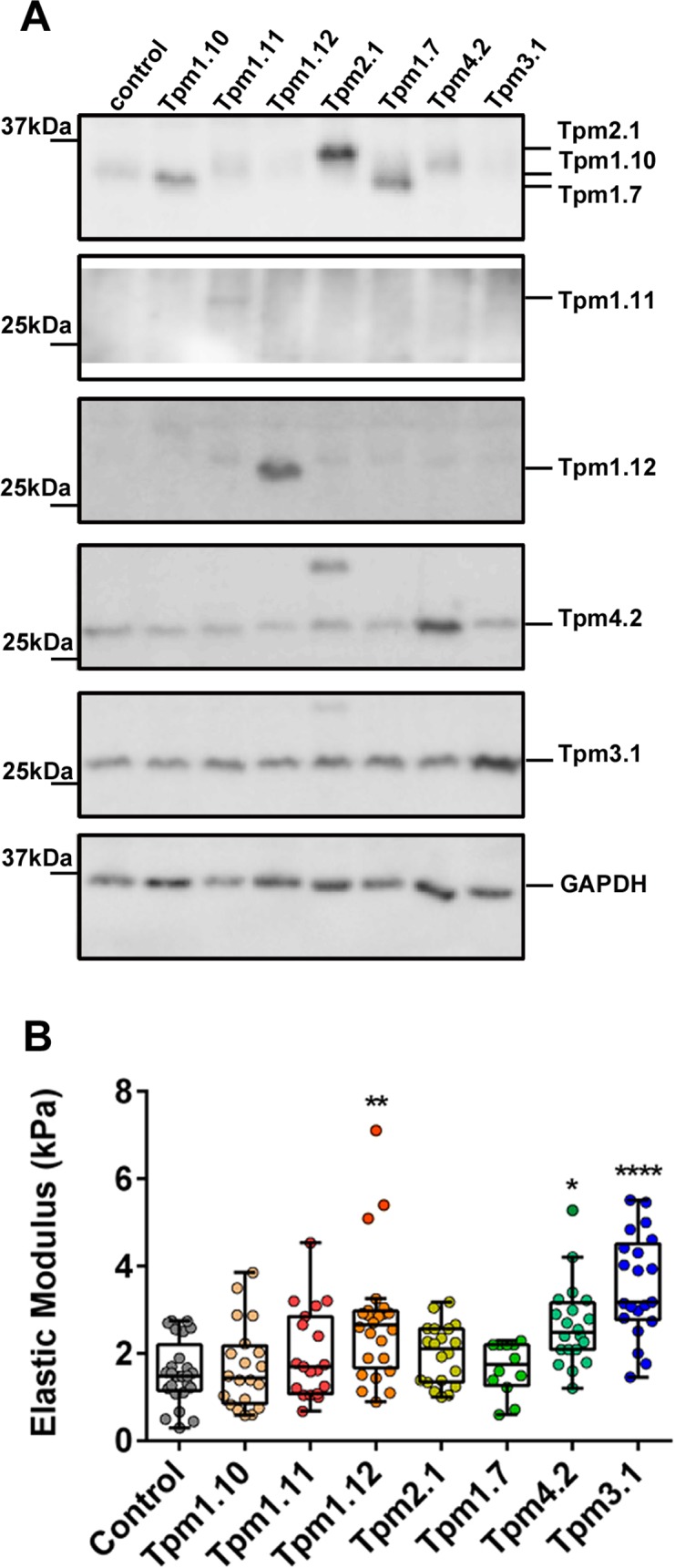
Distinct Tpm isoforms differentially impact on the elastic modulus of the cell. Tpm-overexpressing clones were generated by the stable transfection of Tpm containing vectors. (A) 10 μg of total cellular protein isolated from the Tpm- clones was analysed by SDS-PAGE followed by western blotting. Shown are representative blots probed with the Tm311 (detecting Tpm2.1, Tpm1.10, Tpm1.7), α/9b (Tpm1.11), α/9c (Tpm1.10, Tpm1.12), δ/9d (Tpm4.2), γ/9d (Tpm3.1), and GAPDH antibodies. (B) The elastic (Young) modulus for each Tpm-overexpressing clone was determined. All the data points are presented as box and whisker plots/scatter dots with horizontal line (inside box) indicating median and outliers. 12–25 cells for each clone was measured from *n* = 3 independent experiments. *P<0.05, **P<0.01 compared to control cells. ***P<0.001, ****P<0.0001 compared to control.

It is well established that the mechanical properties of a cell are largely determined by the actin cytoskeleton [3]. However, the studies conducted to date have relied on the use of pharmacological agents such as cytochalasin D or lactrunculin A that are unable to distinguish between the different actin filament populations known to be present within a cell [3,4,6,8,14,15]. In order to gain a better understanding of the molecular mechanism that contributes to the mechanical properties of the cell, we used B35 cells (a neuroblastoma cell line) that overexpress different Tpm isoforms (Fig 1A) known to contribute to the specialization of actin filament networks in mammals and yeast [24,43].

In this paper we used the newly developed nomenclature for Tpms [44]. The isoforms studied herein, Tpm2.1, Tpm1.7, Tpm4.2, Tmp3.1, Tpm1.10, Tpm1.11 and Tpm1.12, correspond to the previous nomenclature of Tm1, Tm3, Tm4, Tm5NM1, TmBr1, TmBr2 and TmBr3.

The elasticity of individual cells was measured by indenting the cells over the nuclear region using an Atomic Force Microscope (AFM) in Peak Force Tapping mode and calculating the Young’s modulus (Elastic modulus kPa). An average of 20 indentations at the plasma membrane directly over the nuclear region was conducted. Considering the differences in cell type, culturing conditions and parameters utilized to obtain a modulus value, our measurements for the control B35 neuroblastoma cells, 1.58 ± 0.15 kPa (Fig 1B), fell within the range previously reported for various cancer cells types irrespective of the cancer type [45]. The stable overexpression of different Tpm isoforms did lead to differential changes in the cell’s elastic modulus. While we observed diverse cell morphologies in the topographical analysis of B35 cells, overexpressing different Tpm isoforms (S2 Fig), we found a significant increase in the cell’s modulus in the Tpm1.12, Tpm4.2, and Tpm3.1-overexpressing cells (2.73±0.29, 2.65±0.21 and 3.56±0.26 kPa, *P*<0.01, *P*<0.05 and *P*<0.0001 respectively) relative to control cells, (1.58 ± 0.15 kPa) (Fig 1B). In contrast, overexpression of Tpm1.10, Tpm1.11, Tpm2.1 and Tpm1.7 lead to no significant changes in the modulus (Fig 1B). We confirmed that repeated indentations lead to no permanent damage or plastic deformation induced by the AFM probe as no significant changes in the elastic modulus were detected over multiple indentations (S3 Fig).

**Fig 2:**
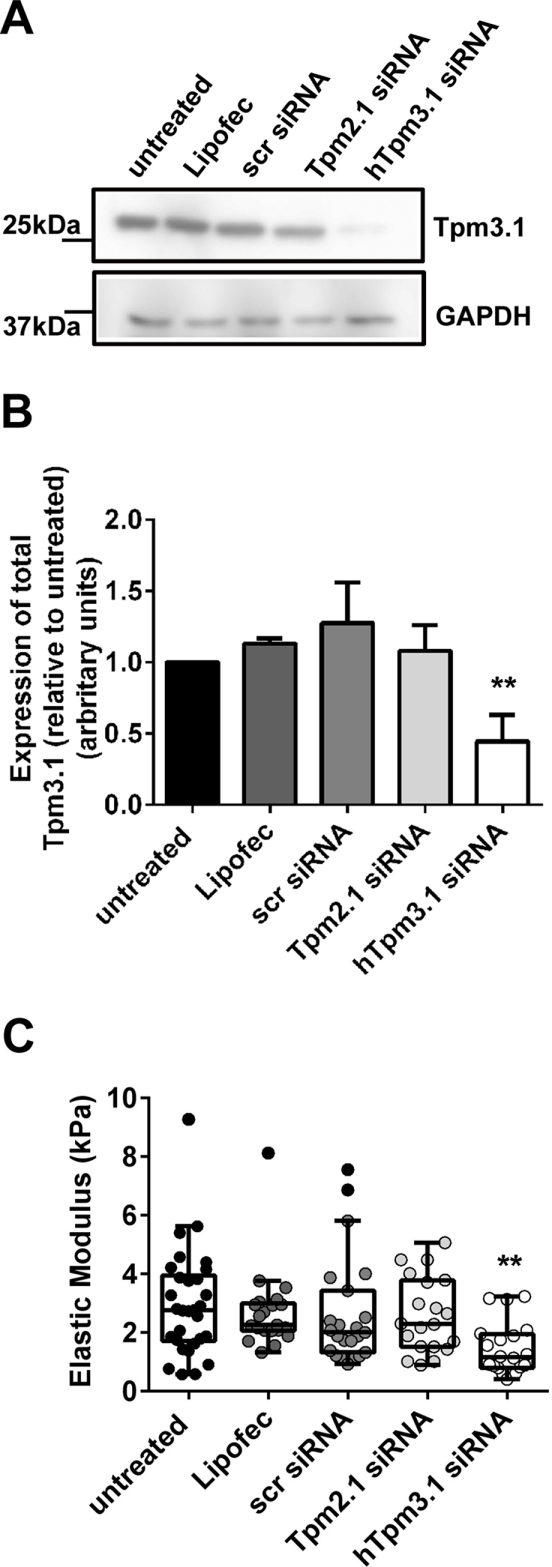
Tpm3.1 knockdown leads to a decrease in the cell’s elastic modulus. (A) Representative westerns of 10 μg of total cellular protein isolated from the Tpm3.1- overexpressing cells untreated (untreat) or exposed to lipofectamine (Lipofec), scramble siRNA (scRNA), Tpm2.1 siRNA or human Tpm3.1 siRNA probed with the γ/9d (mouse and human Tpm3.1) and GAPDH as loading control. (B) Quantification of the total levels of Tpm3.1 levels, *n* = 3. (C) The elastic modulus for each of the treated Tpm3.1-overexpressing cells was determined. All the data points are presented as box and whisker plots/scatter dots with horizontal line (inside box) indicating median and outliers. ≥ 25 cells for each treatment from *n* = 3 independent experiments. **P<0.01, compared to control.

To determine whether the observed increase in the cell’s elastic modulus, seen primarily in the Tpm3.1-overexpressing cells, was due to the expression of Tpm3.1, siRNA knockdown of the exogenously expressed human Tpm3.1 in these cells was performed. Western blot analysis shows a significant decrease in the expression of Tpm3.1 in the Tpm3.1-overexpressing cells (Fig 2A and 2B). As predicted, knockdown of the exogenous human Tpm3.1 led to a significant decrease in the Young’s modulus (2.64±0.38 scramble siRNA, 1.45±0.2 kPa in the Tpm3.1 knockdown cells) (Fig 2C). This was a Tpm isoform specific effect as knockdown of Tpm2.1 lead to no significant changes in the elastic modulus (Fig 2C).

### Overexpression of Tpm isoforms alters the amount and organization of F-actin

**Fig 3:**
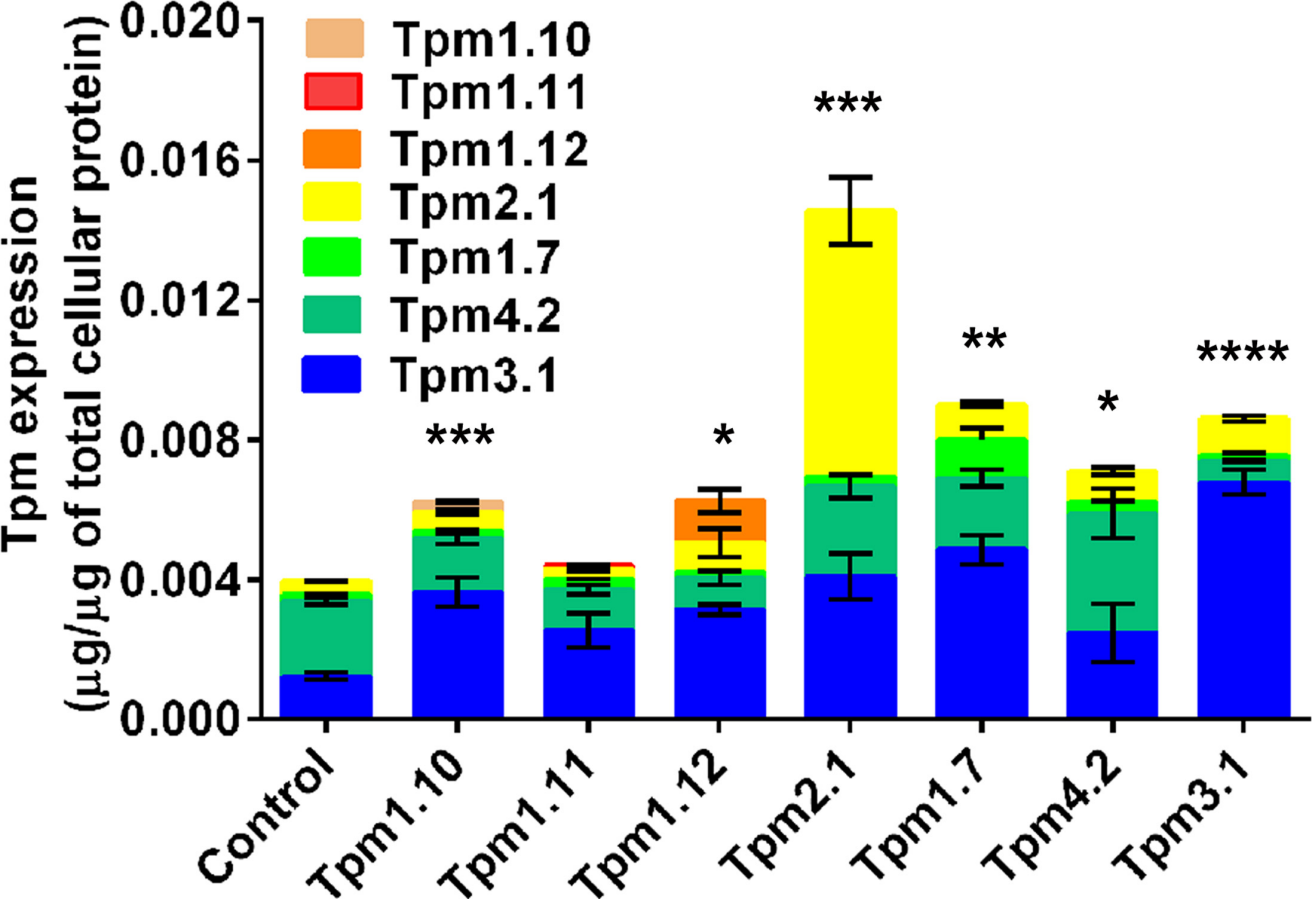
Total Tpm protein levels in the Tpm-overexpressing clones. The total Tpm protein expression levels were determined in each Tpm-overexpressing clone. The expression of each Tpm isoform, evaluated by western blotting, was determined with the use of Tpm recombinant proteins and expressed as μg of Tpm per μg of total cellular protein. The histogram depicts the sum of the most highly enriched Tpm isoforms (Tpm1.6, 1.7, 1.10, 1.11, 1.12, 2.1, 3.1, 4.2). *n* = 4 independent cell lysates, *P<0.05, **P<0.01, ***P<0.001, ****P<0.0001.

To test whether the observed changes in the elastic modulus were simply due to the overexpression of Tpm, an analysis of the total amount of the most enriched Tpm isoforms was undertaken. Total cell lysates together with varying concentrations of Tpm recombinant protein were immunoblotted with different Tpm isoform-specific antibodies. An example of such an analysis is shown in S4 Fig. The bacterial expression system used to generate the recombinant Tpm proteins introduces a six-histidine affinity tag, rendering the recombinant Tpm proteins with a higher molecular weight to that exhibited by the endogenous proteins. We show that all of the Tpm-overexpressing clones, except for the Tpm1.11-overexpressing cells, exhibited significantly elevated levels of Tpm protein, with the Tpm2.1 cells having a 3.7 fold increase in the total amount of Tpm protein expressed relative to that seen in control cells (Fig 3). Moreover, all of the clones displayed differences in the amount of each individual Tpm isoform expressed (see color bars, Fig 3). Thus, the elastic modulus does not track with elevated levels of Tpm expressed in the cells.

**Fig 4:**
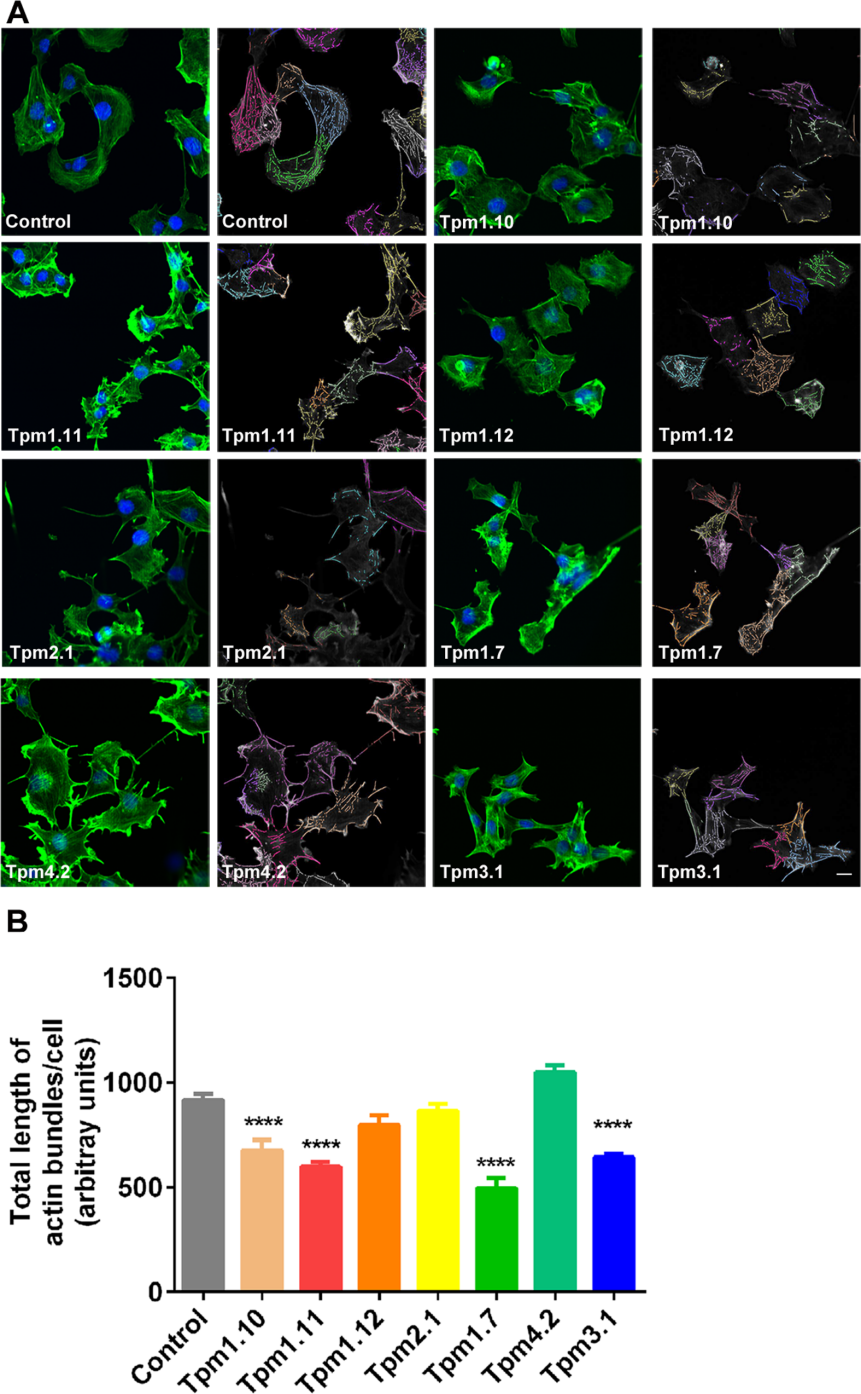
Impact of the different Tpm isoforms on the organization of the actin cytoskeleton. (A) Representative immunofluorescence images of the Tpm-overexpressing B35 clones stained with 488-Atto phalloidin to visualise F-actin (green) and DAPI (blue) for the nucleus and the corresponding colored overlays defining F-actin in the cells. (B) A linear feature detection algorithm was employed to determine total F-actin length/per cell. Each clone was plated in 8 replicates, 6 random fields were imaged and a range of 320 to 590 cells were analysed from *n* = 3 independent experiments. Scale bar, 10 μm. ****P<0.0001.

Studies performed by others have shown a direct correlation between the degree of co-aligned F-actin fibers found in cells, cultured *in vitro* and cell stiffness [46–48]. Since Tpm isoforms play a pivotal role in the stability and the functional specification of actin filaments [24,49], we hypothesized that different Tpm isoforms lead to changes in the F-actin pool and this would correlate with cell stiffness. Two approaches were undertaken to evaluate the F-actin content in the Tpm-overexpressing cells. Firstly, we quantified the organization of the actin cytoskeleton by using a linear detection algorithm to evaluate the organization of the actin cytoskeleton [40] (S5 Fig). The software identifies cells with the use of the nuclear stain DAPI, and filament bundles marked with phalloidin are color coded for each individual cell (Fig 4A). The total length of linear structures representing actin bundles was measured per cell (Fig 4B). Marked differences between the different Tpm-overexpressing clones were observed with a significant decrease in the total length of actin bundles/cell seen in the Tpm1.10, Tpm1.11, Tpm1.7 and Tpm3.1 cells (Fig 4B).

**Fig 5:**
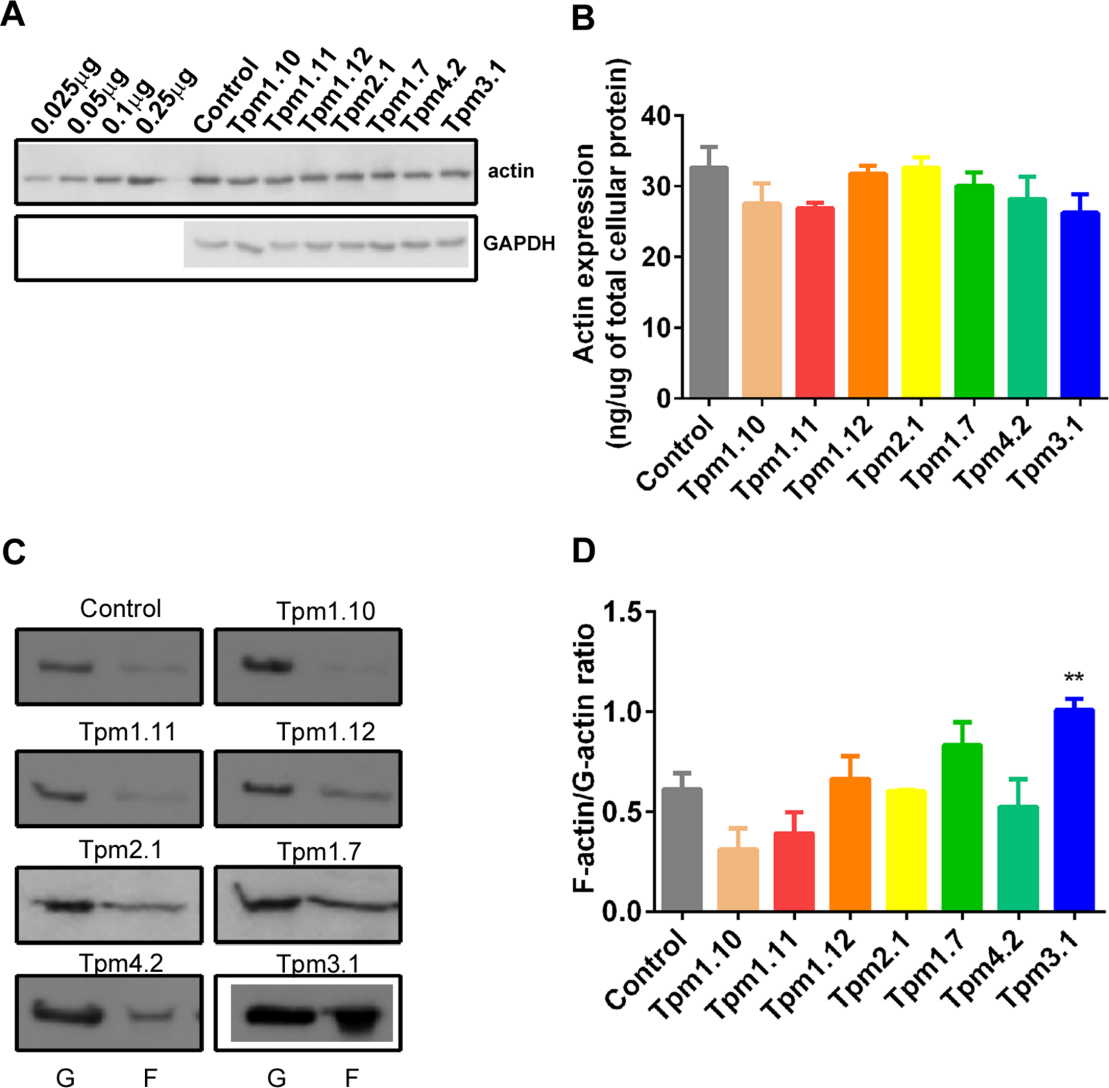
Overexpression of Tpm3.1 alters the F/G actin ratio. (A) 10 μg of total cellular protein from the B35 clones together with skeletal actin protein (0.025, 0.05, 0.1, 0.25 μg) was analysed by SDS-PAGE followed by western blotting and probe with an actin antibody and GAPDH as loading control. A representative blot is shown from *n* = 3 independent experiments. (B) Actin levels were determined by densitometry and expressed as ng of actin per μg of total cellular protein, *n* = 3. (C) Biochemical fractionation of the G and F-actin pools from the different Tpm-overexpressing B35 clones were performed followed by SDS-PAGE electrophoresis. Shown are representative western blots probed for actin. (D) The F-actin/G-actin ratio was determined by densitometric scan of the blots. Shown are the mean ± SEM, *n* = 5. **P<0.01 compared to control.

Actin filament bundles were further evaluated biochemically by determining the expression of actin and the partitioning of insoluble (F-actin) and soluble (G-actin) actin in the Tpm-overexpressing cells. The amount of actin remained unchanged across all Tpm-overexpressing clones (an average in all clones of 29.5 ± 0.93 ng of actin per μg total protein) (Fig 5A and 5B), constituting ~3% of the protein in the cells, agreeing with previous findings showing 5–10% [50]. This data is consistent with our previous findings showing that overexpression of two cytoskeletal Tpm isoforms, Tpm1.7 and Tpm3.1, in transgenic mice models, had no effect on the overall expression of actin, seen in various different tissues [51]. In contrast, we detected a significant increase in the F-actin/G-actin ratio in the Tpm3.1-overexpressing clone, relative to that seen in control cells (Fig 5C and 5D, 1.01 ± 0.06, respectively, relative to control 0.61 ± 0.08, *P*<0.01), consistent with a Tpm3.1-mediated increase in filamentous actin observed previously in cultured cells [51]. The remaining clones exhibited no significant change in the F/G ratios relative to that seen in control cells. Collectively, these data indicates that overexpression of Tpm does not necessarily translate into an increase in F-actin and that the influence of Tpm expression on the F/G-actin ratio is Tpm isoform-dependent.

Microtubules have been reported to have minimal contribution to the elastic properties of cells and the contribution of intermediate filaments is small and dependents on substrate stiffness and indentation depth [9,10,52]. To test whether the observed changes in the cell’s elastic modulus (Fig 1B) could be influenced by either of these cytoskeletal polymer systems, total levels of expression of αtubulin and vimentin were determined. The expression levels of αtubulin and vimentin were found to be unchanged across all the Tpm-overexpressing clones relative to the control (S6 Fig).

Taken together these results demonstrate that the elastic modulus of the cell is mediated by Tpm in an isoform-specific manner with the Tpm1.12, Tpm4.2, and Tpm3.1-overexpressing cells showing an increase in the elastic properties relative to control cells. The observed cellular elastic modulus is independent on the levels of expression of αtubulin or vimentin.

### Tpm impacts on the cell’s mechanical properties via non-muscle myosin II

**Fig 6:**
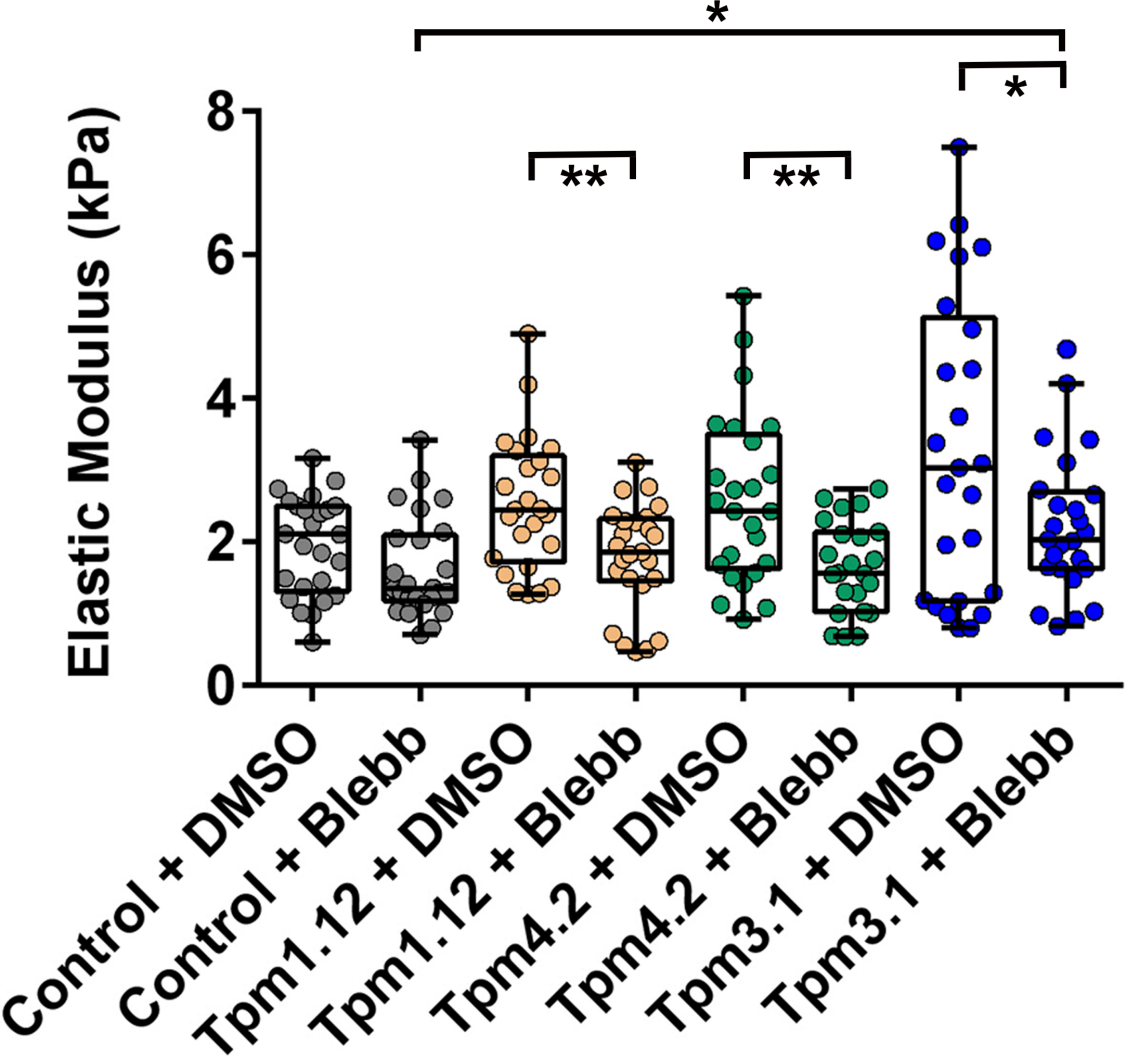
Effect of blebbistatin on the cell’s elastic properties. The elastic (Young) modulus for the Tpm1.12, Tpm4.2 and Tpm3.1-overexpressing clones was determined following DMSO or 50 μM of blebbistatin treatment for 30 mins. All data points are presented as box and whisker plots/scatter dots with horizontal line (inside box) indicating median and outliers. 23–25 cells for each clone was measured from *n* = 3 independent experiments. *P<0.05, **P<0.01 compared to control cells.

The mechanical properties of cells have been shown to be determined by cytoskeletal tension generated by non-muscle myosin II, which links actin filaments and generates contractile force leading to contraction of the actin cytoskeleton. To test whether the observed increase in cell stiffness seen in the Tpm1.12, Tpm4.2 and Tpm3.1 cells requires non-muscle myosin II, cells were exposed to blebbistatin, a specific inhibitor of the interaction of actin with non-muscle myosin II [53]. Blebbistatin has previously been shown to decrease the elastic modulus of cells cultured *in vitro*, isolated rat glomeruli, in salivary glands *in situ* excised from mice [54–56] and of individual actin stress fibers in living endothelial cells [57]. Phase contrast microscopy showed that treatment of the cells with blebblistatin resulted in the emergence of long extensions resembling neurite outgrowth (S7 Fig arrows). Similar structures have been reported following the treatment of primary cultures of chicken forebrain neurons with blebbistatin [58]. Unexpectedly, no significant difference in the elastic modulus was detected in the control B35 cells following exposure to blebbistatin as compared to the vehicle treatment (Fig 6). Conversely, a significant decrease in the elastic modulus was seen in the Tpm1.12, Tpm4.2 and Tpm3.1-overexpressing cells following exposure to blebbistatin as compared to the vehicle (DMSO) (2.53±0.19 to 1.79 ± 0.15, *P*<0.01, 2.59±0.24 to 1.66±0.13, *P*<0.01, and 3.3±0.42 to 2.2±0.19 kPa, *P*<0.05, respectively) (Fig 6 and S1 Table). The Tpm overexpressing cells exhibited a decrease in the elastic modulus following exposure to blebbistatin with 29, 36 and 33% seen in the Tpm1.12, Tpm4.2 and Tpm3.1-overexpressing cells, respectively, as compared to that seen with the DMSO alone. Collectively, these results show that the observed increase in cell stiffness is mediated via intracellular tension generated by non-muscle myosin II.

### Tpm3.1 alters the elastic modulus of adipose tissue

**Fig 7:**
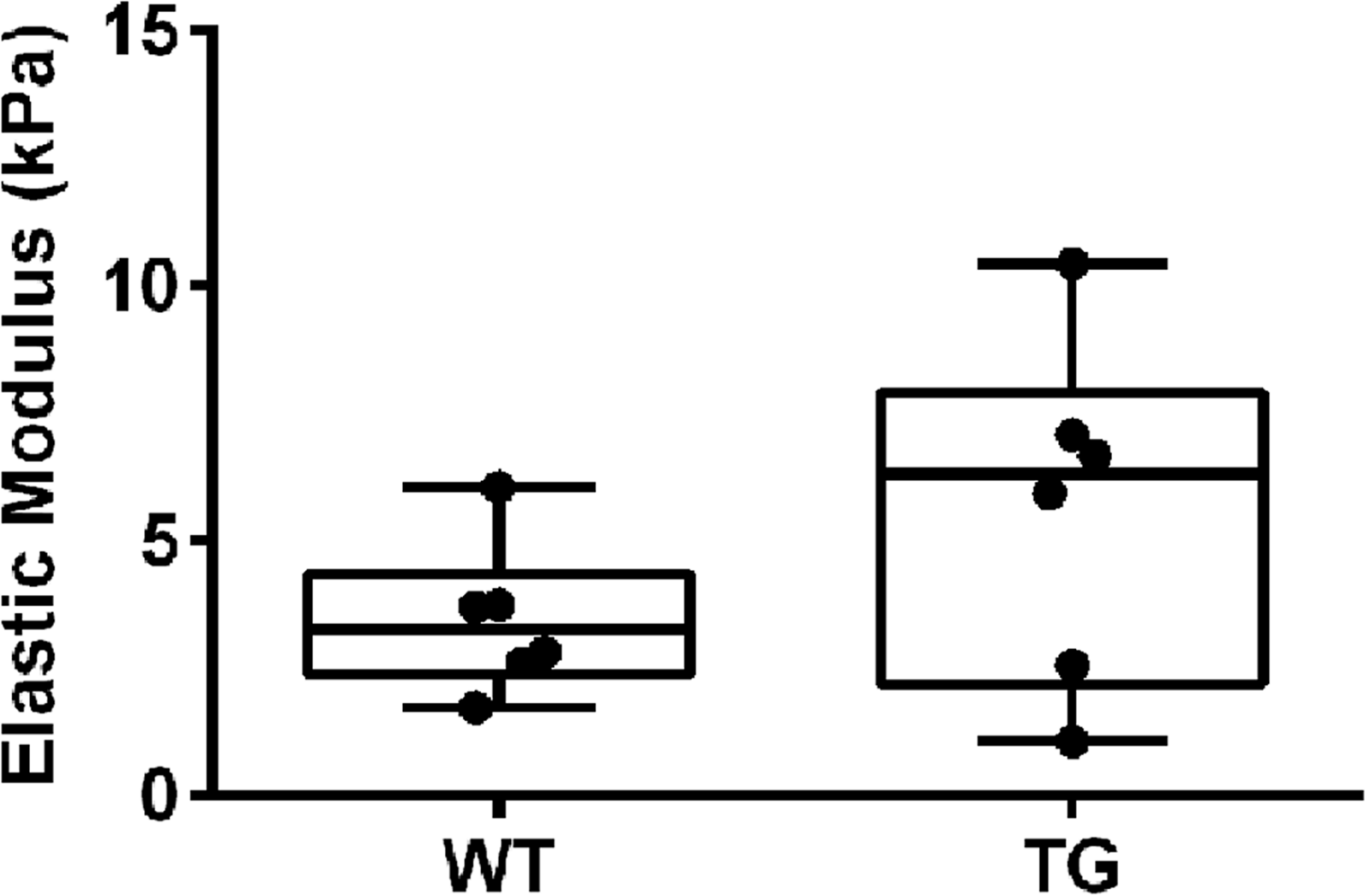
Nanomechanical properties of mouse adipose tissue from Tpm3.1 transgenic mice. Elastic modulus values of epididymal fat isolated from the Tpm3.1-overexpressing TG and control (WT) mice. A total of 6 mice per group were tested, 10–50 indentation points per area (area was 30 μm^2^) was conducted and 5–10 areas per epididymal fat pad (both left and right) were employed. Data is visualised as a box plot showing the median with the interquartile range and all the data points. Data points represent the mean of all indentations conducted per animal. A nonparametric, Mann-Whitney test shows no statistical differences between WT and TG samples.

Different tissues are known to have well-defined elastic moduli ranging from soft tissues such as fat to those with much greater stiffness like cartilage and bone [59]. To test whether the significant increase in the elastic modulus mediated by Tpm3.1 is recapitulated in cells *in situ* we measured the mechanical properties of fresh tissues isolated from Tpm3.1-overexpressing transgenic mice (TG) mice. In order to avoid a wide variation in the elastic modulus, we assessed the mechanical properties of epididymal fat, a tissue known to be relative homogenous in terms of the cell types present. Epididymal fat pads from the TG mice have previously been shown to have a 3-fold increase in total Tpm3.1 levels (A. J. Kee and P. Gunning, publication under review). Multiple indentations in Peak Force Tapping in various regions were conducted per pad and both the left and right pads were used. We obtained measurements that are in agreement with those previously reported for similar mammalian soft tissues, measured by nanoindentation [60]. Although not significantly different we did observe a trend towards an increase in the elastic modulus in the TG fat pads relative to the WT (Fig 7).

## Discussion

### Contribution of Tpm to the mechanical properties of cells

Tpm isoforms display distinct spatial and temporal subcellular distributions and are known to contribute to the functional diversity of actin filaments by regulating the actin binding proteins and myosin motors that are capable of associating with the filaments [21–25,31]. Our finding that altered expression of different Tpm isoforms differentially alters the elastic modulus of cells further supports the existence of different actin filament networks defined by Tpm isoforms. Even cytochalasin D has been shown to give rise to a heterogeneous response in the elastic modulus when exposed to fibroblasts as opposed to erythrocytes, which are more homogenous in their response [36]. This suggests that within fibroblasts different actin filament networks are present which respond differently to the effects of cytochalasin D. Our previous work has demonstrated that Tpm3.1-containing actin filaments are more resistant to the effects of cytochalasin D as compared to other Tpm isoform-containing actin filaments [32,61].

**Fig 8:**
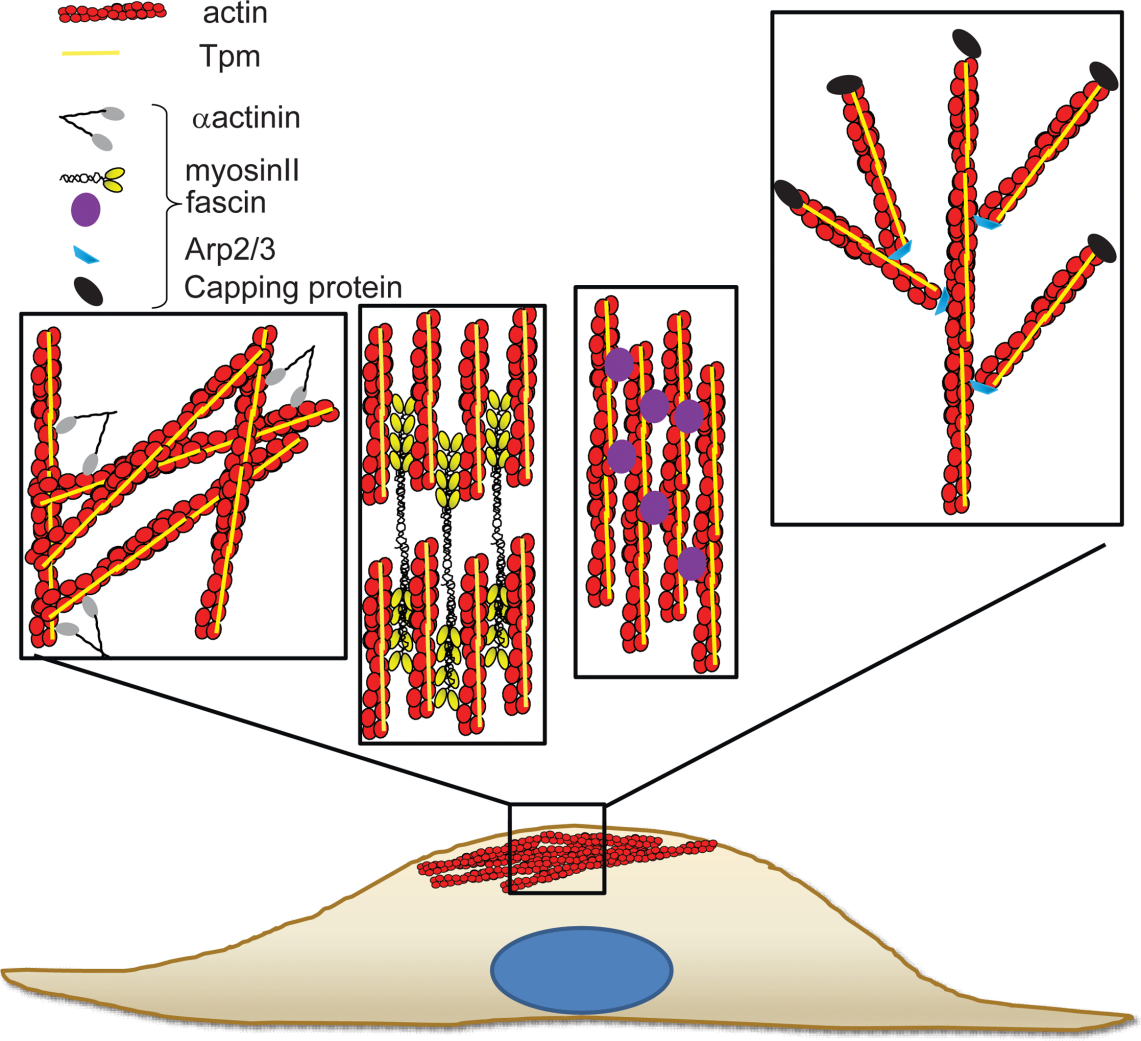
Model proposing the potential impact of distinct actin filament populations on cell stiffness. We propose that Tpm can define different actin filament populations by dictating the recruitment of different actin binding proteins. Shown are crosslinked filaments associating with αactinin, tension bearing filaments with myosinII, bundles with fascin and branched actin filaments with Arp2/3 and Capping protein. The distinct organisation and functional properties of these Tpm-containing cortical actin filaments impact on cell stiffness.

The cell’s mechanical properties have been largely attributed to the actin cytoskeleton. However, the precise mechanism at the level of the molecular composition and/or spatial organization of the actin filaments remains poorly defined. Previous studies have repeatedly demonstrated that formation of actin stress fibers tracks with cell stiffness [62,63]. However, other studies do show that neither stress fibers nor the F-actin content alone was predictive of cell stiffness or contractility [64,65]. Hence, these authors postulated that the spatial organization of the actin cytoskeleton in terms of the degree of actin microfilament cross-linking, which can generate a dense meshwork of filaments, is a key determinant of cellular mechanics. This observation is clearly seen in reconstituted F-actin filaments *in vitro*, stabilized by the addition of cross-linking actin binding proteins which led to the generation of F-actin meshworks with increased elasticity [66–70]. Together, these studies are in agreement with our data showing that cell stiffness is not entirely related to the F-actin content, detected both by quantitation of phalloidin stained F-actin in cells and biochemically (F/G ratio) (Fig 4, Fig 5C and 5D and S2 Table). This is clearly seen in Tpm1.12 and Tpm4.2-overexpressing cells which display an elevated elastic modulus with no significant changes detected in the F/G ratio (Fig 1B, Fig 5C and 5D and S2 Table) or total F-actin content detected by immunostaining (Fig 4, S2 Table). Hence, we propose, as depicted in the model seen in Fig 8, that different Tpm-containing actin filament populations drive different biomechanical outcomes via differences in their recruitment of different actin binding proteins [21,23,31]. It should be noted, however, that each tropomyosin for which there is data, can engage in multiple cellular functions by contributing to the formation of different actin filament networks. Thus, Tpm isoforms may be present in more than one actin filament network defined spatially and temporally by the molecular composition of the subcellular compartment [24,71].

### Tpm and myosin and the regulation of cell stiffness

Evidence from various studies using different cell model systems, shows that cortical actin stiffness is mediated by the actomyosin cytoskeleton [55,62,72–74]. A notable finding from our studies is the differential impact on cell stiffness by the different Tpm isoforms mediated via intracellular tension generated by non-muscle myosin II (Fig 6). Biochemical and cellular studies support the concept that myosin motor activity is differentially sensitive to specific Tpm isoforms. Studies in yeast and mammals show that Tpm inhibits the activity of myosin-I (Myo1p in yeast, Myo1c in mammals) but enhances that of myosin-Vs and makes the motor more processive [75–78]. The use of an *in vitro* motility assay to measure isometric force of the actomyosin interaction demonstrates that different Tpm isoforms have differing effects on the motility and ATPase activities of nonmuscle myosin isoforms, IIA, IIB and IIC [79]. Moreover, we have previously shown that Tpm3.1-containing actin filaments have a higher specificity for myosin IIA [21]. Whether the differences between isoforms in their interaction with myosin II account entirely for their impact on cell stiffness remains to be determined.

### Tpm may contribute to the stiffness of fat tissue *in situ*

Adipocyte, brain and pancreas are some of the softest tissues found in mammals [60]. Our data are consistent with these studies with our measurements ranging between 2.81 and 10.44 kPA (Fig 7). Even though a significant difference in the elastic modulus of the fat pads from the WT and TG was not detected, a trend towards elevated values was seen in the TG fat pads. This suggests that the observations made in the Tpm3.1-overexpressing B35 cells could potentially be recapitulated in cells *in situ*. The trend towards an increase in the elastic modulus may in part involve an increase in the F/G actin as seen in the Tpm3.1-overexpressing B35 cells (Fig 5C and 5D). Quantitation of the intensity of phalloidin staining in histological sections of fat pads showed that in the TG mice there is indeed an increase in the intensity of staining, confirming an increase in F-actin (A. J. Kee and P. Gunning, publication under review).

## Conclusion

The impact of Tpm isoforms on cell stiffness aligns with the increasing realization that Tpm isoforms define the functional properties of actin filaments at multiple levels [80]. This validation between isoforms provides strong evidence that the determination of cell stiffness is not simply a function of actin filament levels per se but also involves higher levels of organization and requires myosin II activity.

## Supporting Information

S1 Fig Force-separation curves generated using AFM nanoindentation-type under Peak Force tapping mode. **R**epresentative retraction portion of the force curves for a (A) control and (B) Tpm3.1 cells. The adhesion between the probe and the sample has been considered for the analysis and is depicted as the negative portion of the graphs. The Young’s modulus is extracted from the linear region of the force curves using the Sneddon fit [34].
(TIF)

S2 Fig AFM topographical images of the Tpm-overexpressing B35 cells. The entire cell was scanned under PeakForce Tapping mode at a resolution of 512 × 512 pixel. Arrow indicates prominent stress fibers seen in the Tpm3.1-overexpressing cells. Scale bar, 10 μm.
(TIF)

S3 Fig Multiple indentation measurements lead to minimal impact on the cell’s elastic modulus. The Tpm-overexpressing B35 clones were indented at least 15 times and the elastic modulus determined.
(TIF)

S4 Fig An example of the analysis of the amount of Tpm4.2 protein expressed in the control and Tpm4.2 B35 clones. Representative western blot of 0.005, 0.01, 0.025, 0.0.5, 0.075 and 0.1 μg of recombinant Tpm4.2 protein and duplicate samples of 10 μg of total protein cell lysates (n = 2 lysates) isolated from the control and Tpm4.2-overexpressing cells. Blots were probed with the δ/9d antibody to detect Tpm4.2 and Ponceau red stain was used as loading control.
(TIF)

S5 Fig High content image analysis. (A) A representative immunofluorescent image of control B35 cells stained with ATTO-488 phalloidin for F-actin (green) and DAPI for the nucleus (blue). (B) The image analysis software detects all nuclei, color codes them and (C) total cell area is assigned. (D) Filamentous structures defined as bundles of F-actin are detected. Scale bar, 10 μm.
(TIF)

S6 Fig Effects of overexpression of Tpm isoforms on the expression levels of tubulin and vimentin. 10 μg of total cellular protein from the B35 clones was analysed by SDS-PAGE followed by western blotting. Representative blots probed with antibodies to (A) αtubulin and GAPDH as loading control and (C) vimentin and GAPDH as loading control. (B, D) Corresponding quantitation following the densitometry scan of the blots, expressed as a percentage of the levels seen in the control B35 cells set at 100%. Shown are the mean ± SEM, *n* = 3 independently isolated cell lysates.
(TIF)

S7 Fig Blebbistatin treatment leads to morphological alterations. Phase-contrast micrographs of Tpm1.12, Tpm4.2 and Tpm3.1-overexpressing cells treated with (A, B, C, D) vehicle alone (DMSO) or (E, F, G, H) 50 μM blebbistatin for 30 min. Scale bar 10 μM.
(TIF)

S1 Table The effect of blebbistatin on the cell’s elastic properties. Cells were treated with DMSO or 50 μM of blebbistatin for 30 min and the elastic modulus determined by indenting the cells in Peak Force Tapping mode, according to the parameters described in Materials and Methods. 23–25 cells for each clone from *n* = 3 independent experiments. ^a^Data shown represents the mean ± SEM with P<0.01 compared to Tpm1.12 + DMSO. ^b^Data shown represents the mean ± SEM with P<0.01 compared to Tpm4.2 + DMSO. ^c^Data shown represents the mean ± SEM with P<0.05 compared to Tpm3.1 + DMSO.
(DOCX)

S2 Table Comparison of the elastic modulus with Tpm expression and F-actin quantitation. Above Table is a summary of the data presented in Figs 1*B*, 2, 3 and 4. Highlighted in bold italics are the parameters found to be different relative to control cells. ^a^ *P*<0.05, ^b^ *P*<0.01, ^c^ *P*<0.001 and ^d^ *P*<0.0001 compared to control.
(DOCX)
