# Supplementary figures and images for: Cell Elasticity Is Regulated by the Tropomyosin Isoform Composition of the Actin Cytoskeleton
Source: PLoS One. 2015 May 15;10(5):e0126214. doi: 10.1371/journal.pone.0126214 (PMC4433179; doi:10.1371/journal.pone.0126214)

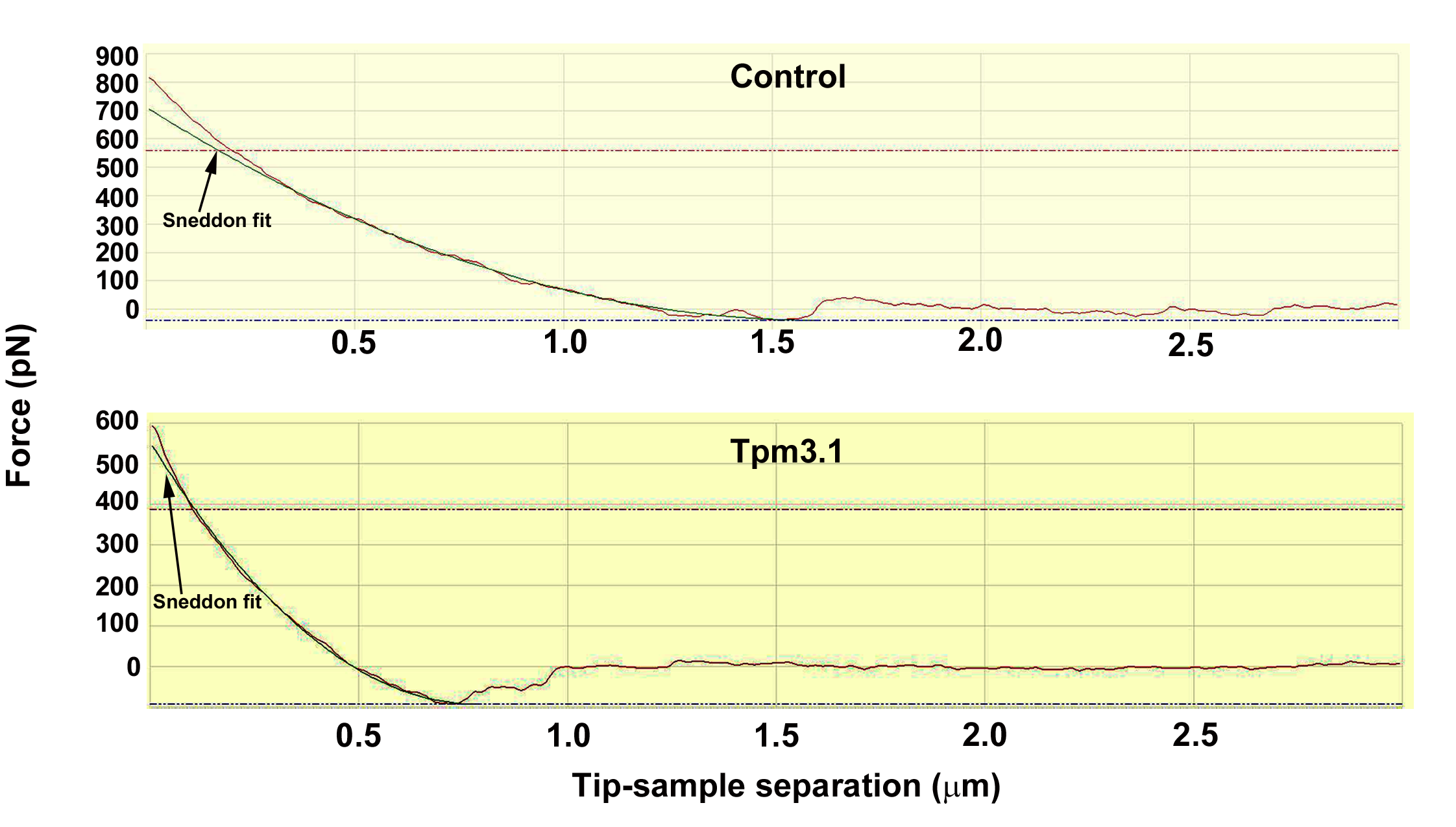

Supplement: S1 Fig — Representative retraction portion of the force curves for a (A) control and (B) Tpm3.1 cells. The adhesion between the probe and the sample has been considered for the analysis and is depicted as the negative portion of the graphs. The Young’s modulus is extracted from the linear region of the force curves using the Sneddon fit [34]. (TIF) [file pone.0126214.s001.tif]

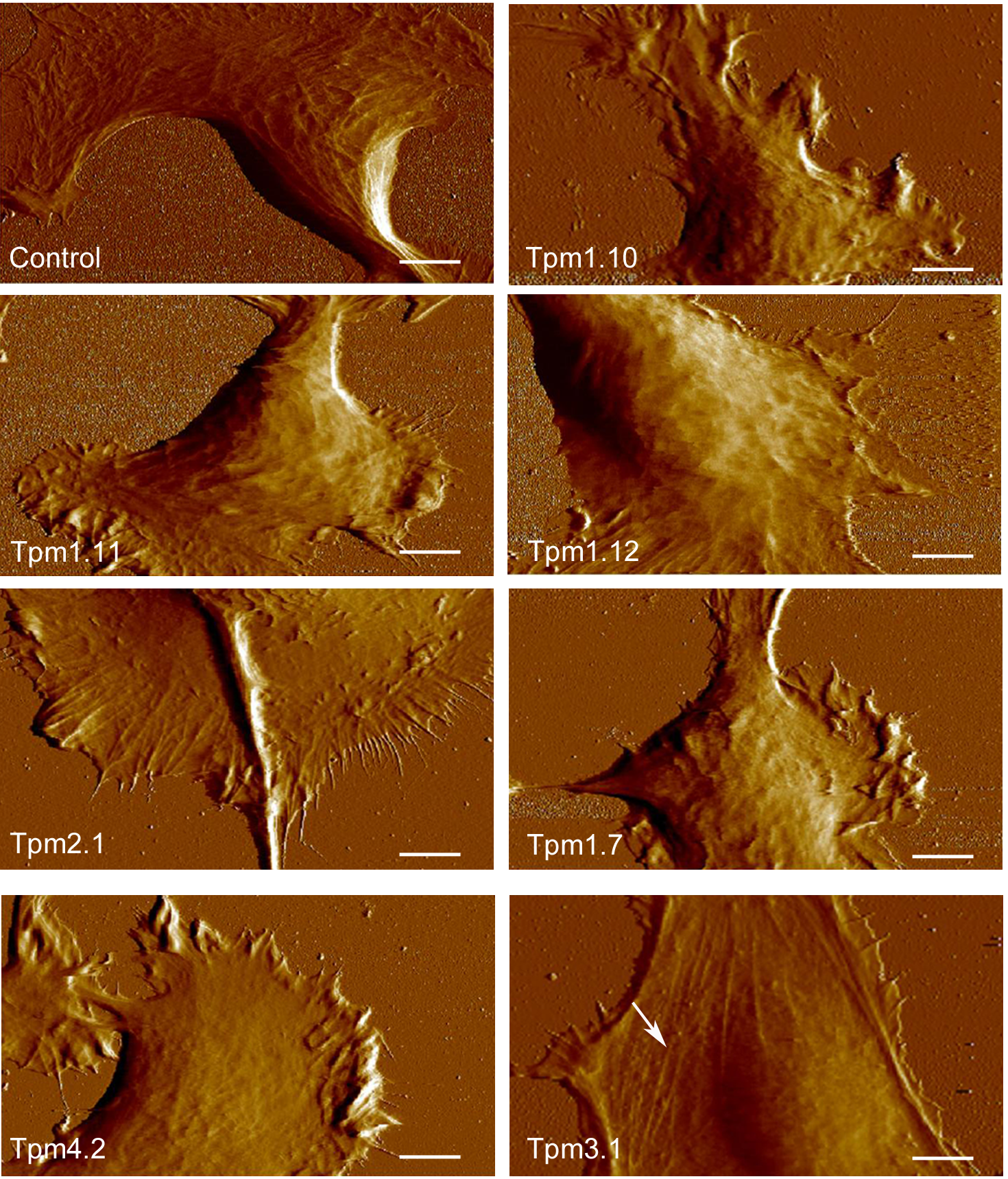

Supplement: S2 Fig — The entire cell was scanned under PeakForce Tapping mode at a resolution of 512 × 512 pixel. Arrow indicates prominent stress fibers seen in the Tpm3.1-overexpressing cells. Scale bar, 10 μm. (TIF) [file pone.0126214.s002.tif]

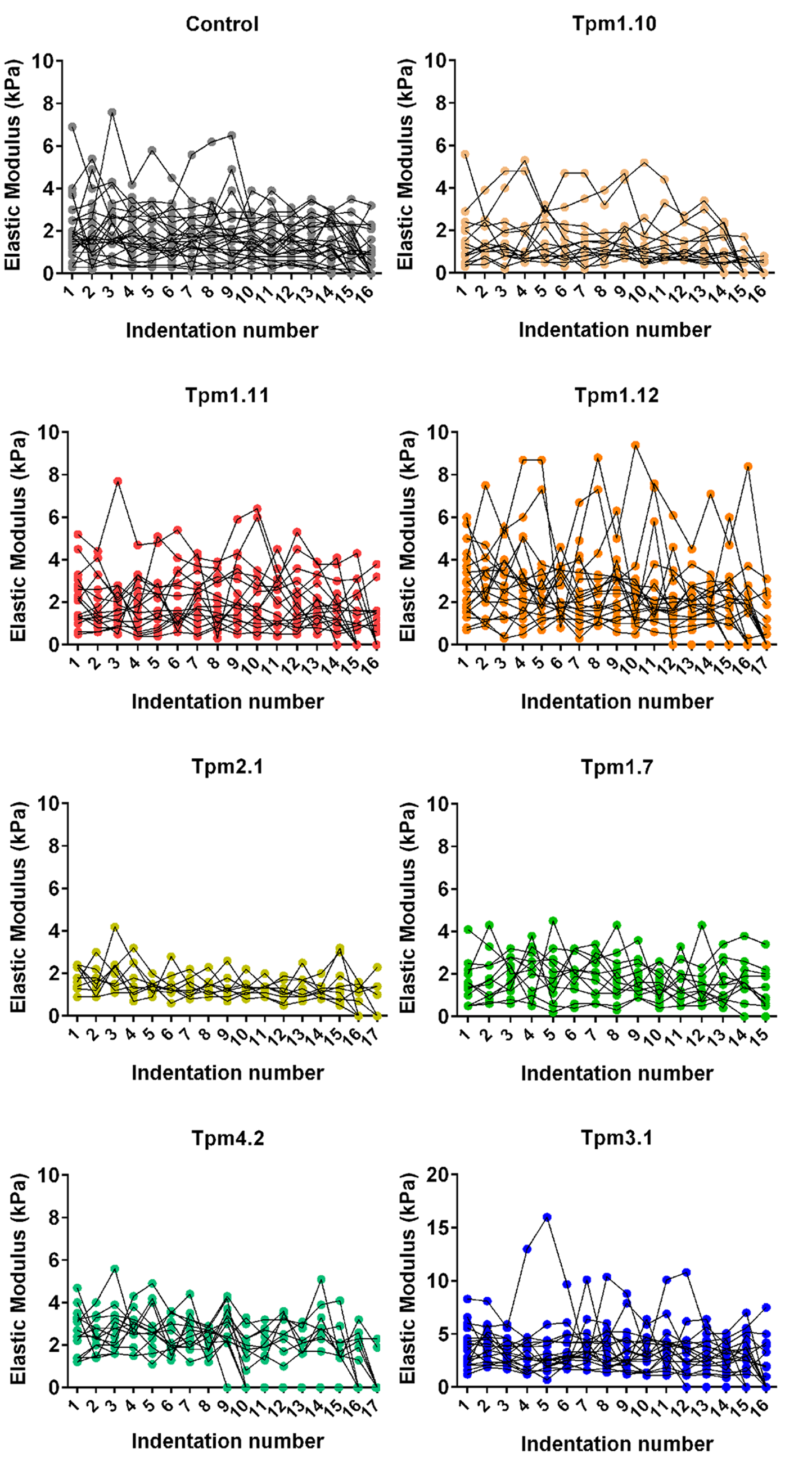

Supplement: S3 Fig — The Tpm-overexpressing B35 clones were indented at least 15 times and the elastic modulus determined. (TIF) [file pone.0126214.s003.tif]

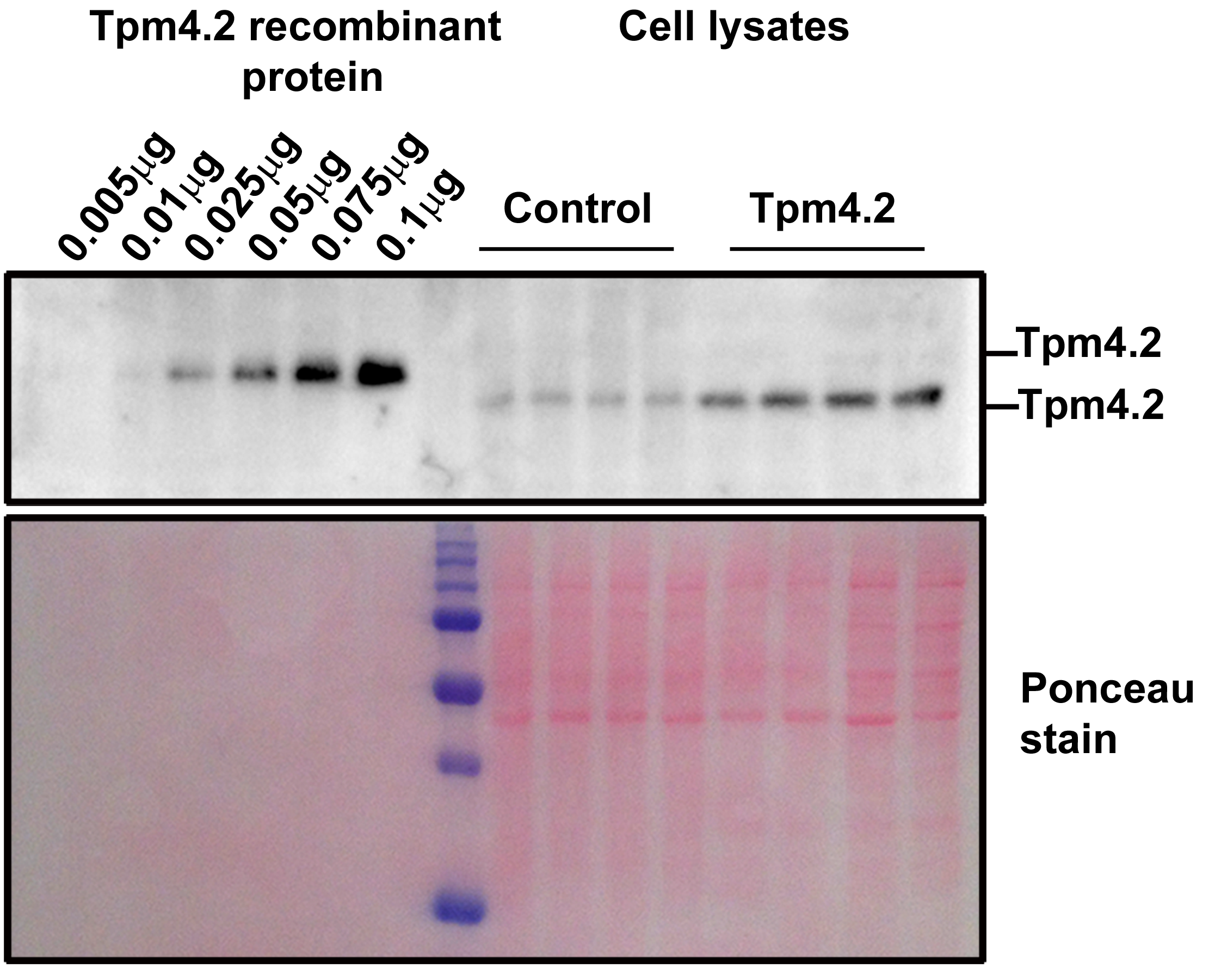

Supplement: S4 Fig — Representative western blot of 0.005, 0.01, 0.025, 0.0.5, 0.075 and 0.1 μg of recombinant Tpm4.2 protein and duplicate samples of 10 μg of total protein cell lysates (n = 2 lysates) isolated from the control and Tpm4.2-overexpressing cells. Blots were probed with the δ/9d antibody to detect Tpm4.2 and Ponceau red stain was used as loading control. (TIF) [file pone.0126214.s004.tif]

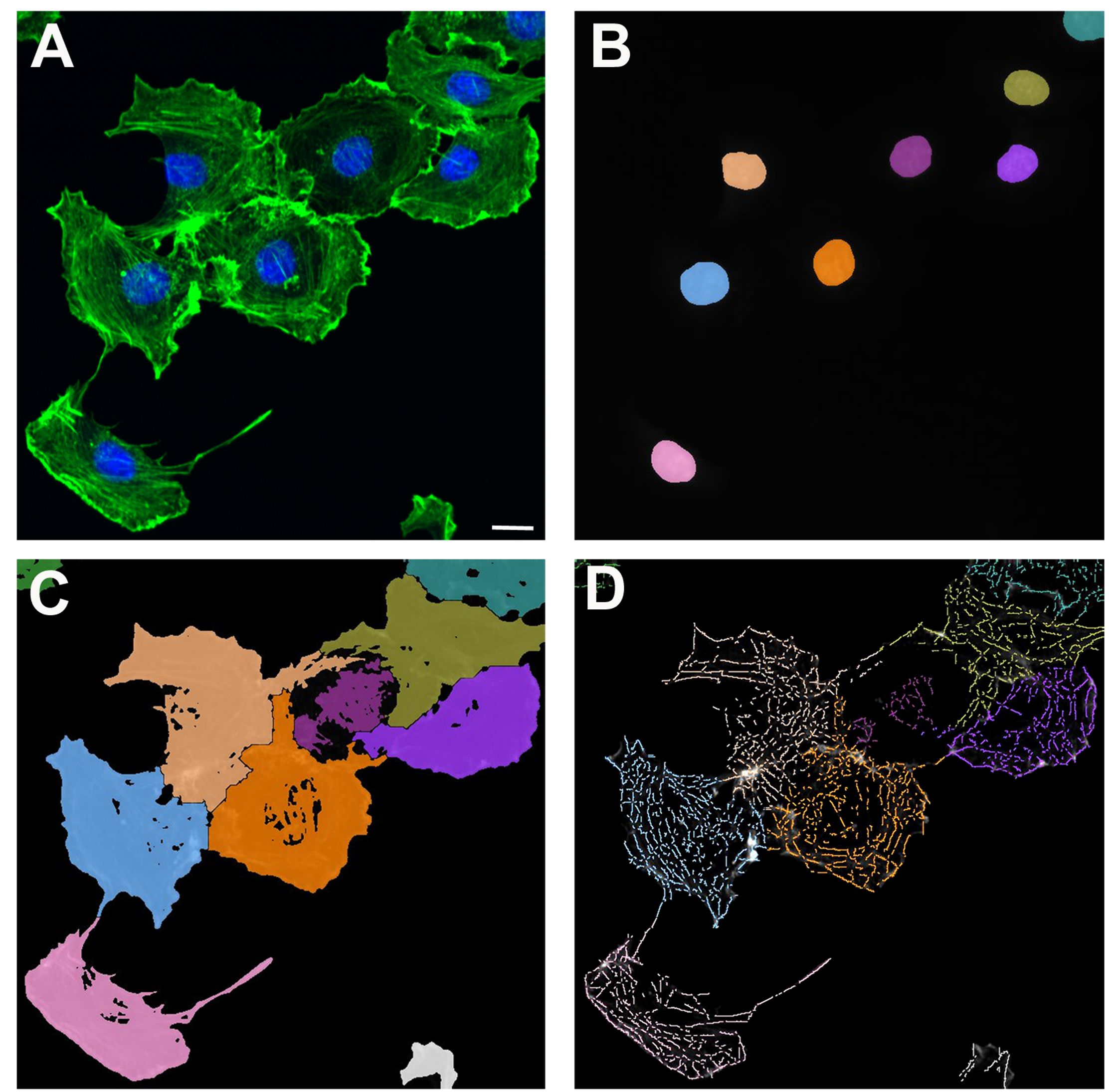

Supplement: S5 Fig — (A) A representative immunofluorescent image of control B35 cells stained with ATTO-488 phalloidin for F-actin (green) and DAPI for the nucleus (blue). (B) The image analysis software detects all nuclei, color codes them and (C) total cell area is assigned. (D) Filamentous structures defined as bundles of F-actin are detected. Scale bar, 10 μm. (TIF) [file pone.0126214.s005.tif]

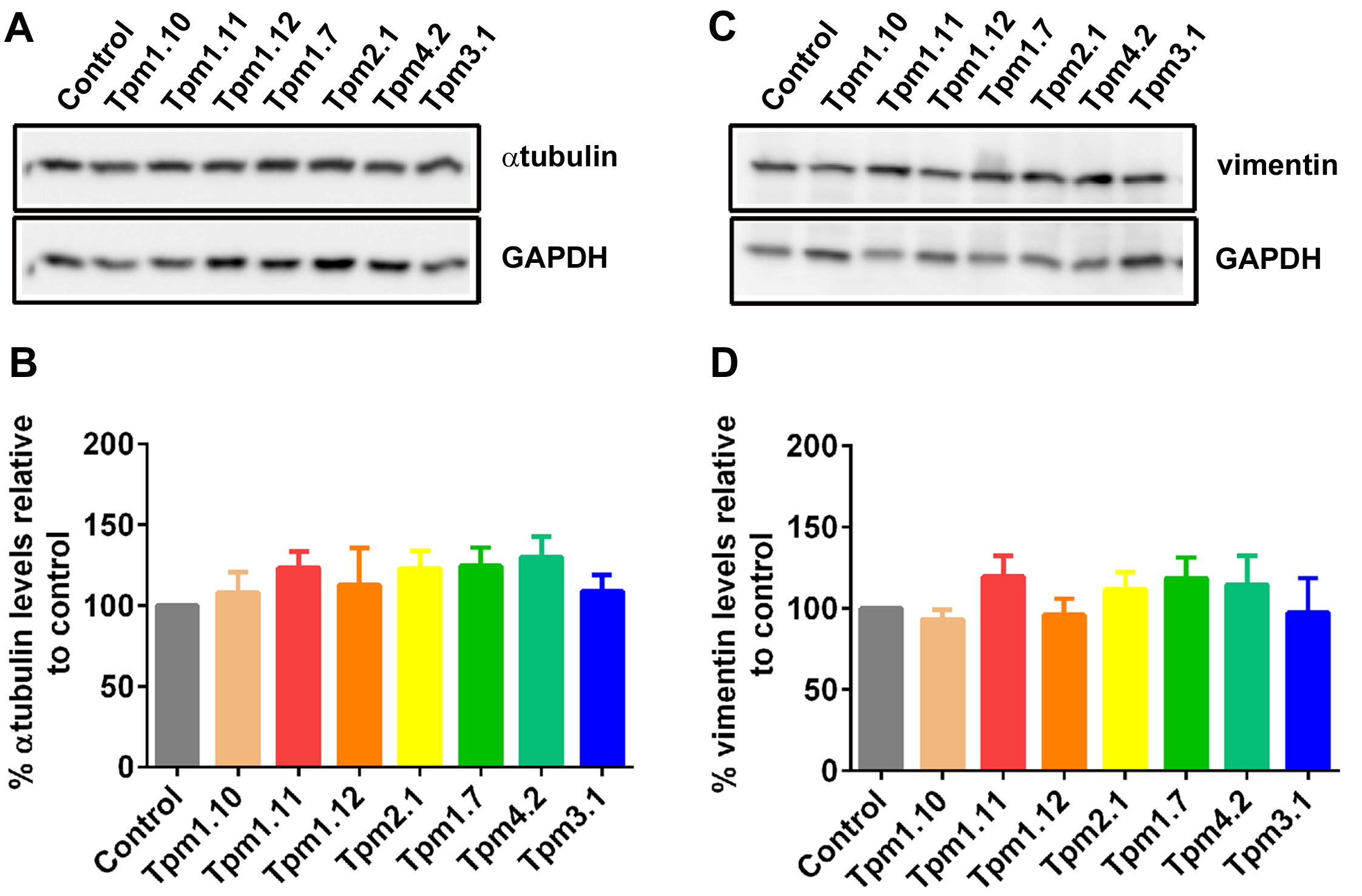

Supplement: S6 Fig — 10 μg of total cellular protein from the B35 clones was analysed by SDS-PAGE followed by western blotting. Representative blots probed with antibodies to (A) αtubulin and GAPDH as loading control and (C) vimentin and GAPDH as loading control. (B, D) Corresponding quantitation following the densitometry scan of the blots, expressed as a percentage of the levels seen in the control B35 cells set at 100%. Shown are the mean ± SEM, n = 3 independently isolated cell lysates. (TIF) [file pone.0126214.s006.tif]

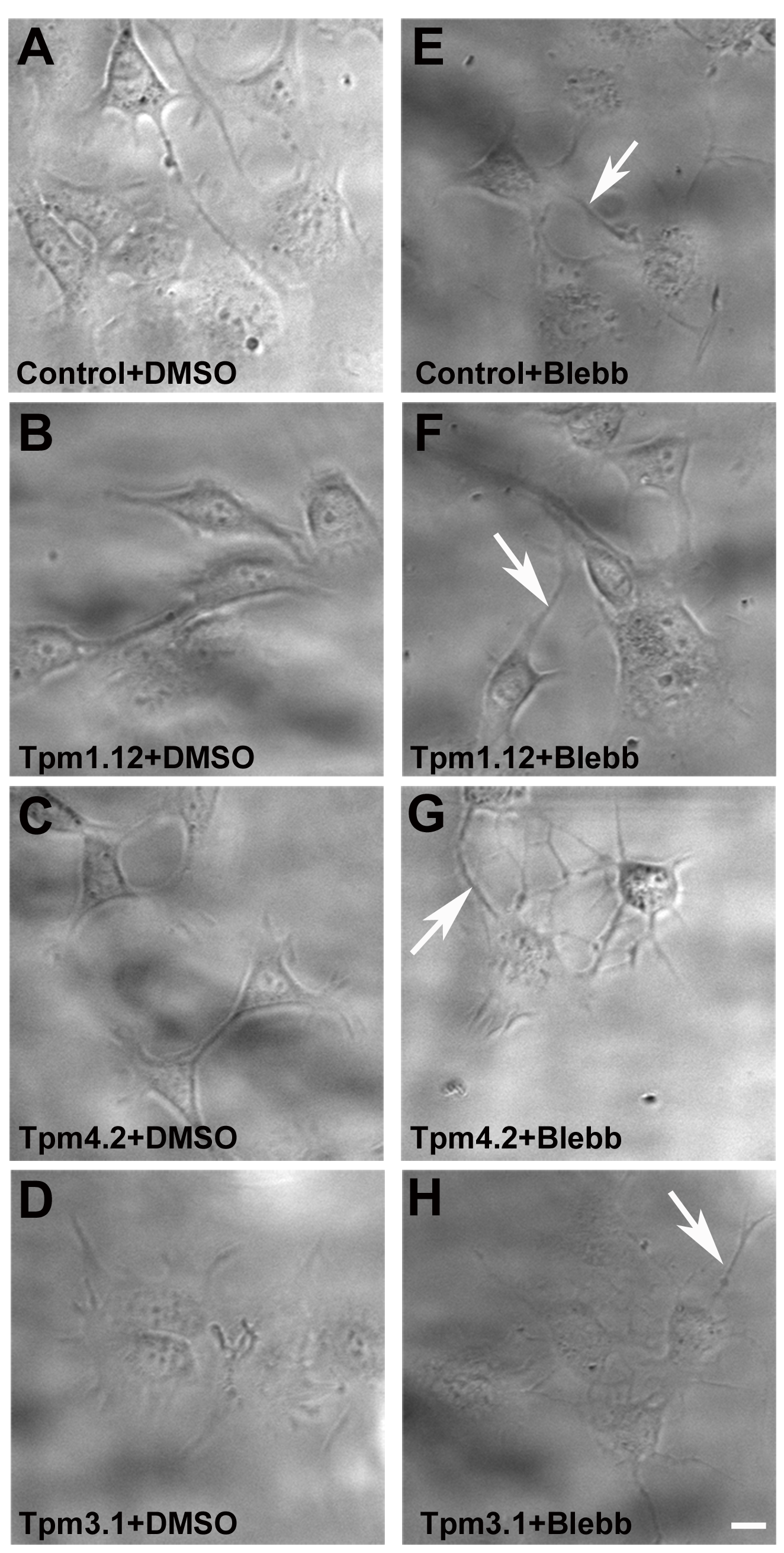

Supplement: S7 Fig — Phase-contrast micrographs of Tpm1.12, Tpm4.2 and Tpm3.1-overexpressing cells treated with (A, B, C, D) vehicle alone (DMSO) or (E, F, G, H) 50 μM blebbistatin for 30 min. Scale bar 10 μM. (TIF) [file pone.0126214.s007.tif]
