# Supplementary material for: Cell Elasticity Is Regulated by the Tropomyosin Isoform Composition of the Actin Cytoskeleton
Source: PLoS One. 2015 May 15;10(5):e0126214. doi: 10.1371/journal.pone.0126214 (PMC4433179; doi:10.1371/journal.pone.0126214)
Supplement: S1 Table — Cells were treated with DMSO or 50 μM of blebbistatin for 30 min and the elastic modulus determined by indenting the cells in Peak Force Tapping mode, according to the parameters described in Materials and Methods. 23–25 cells for each clone from n = 3 independent experiments. aData shown represents the mean ± SEM with P<0.01 compared to Tpm1.12 + DMSO. bData shown represents the mean ± SEM with P<0.01 compared to Tpm4.2 + DMSO. cData shown represents the mean ± SEM with P<0.05 compared to Tpm3.1 + DMSO. (DOCX) [file pone.0126214.s008.docx]

S1 Table. The effect of blebbistatin on the cell’s elastic properties.

| B35 clones | Elastic modulus |
| --- | --- |
| Control + DMSO | 1.956± 0.1374 |
| Control + blebb | 1.634± 0.1408 |
| Tpm1.12 + DMSO | 2.529± 0.1863 |
| Tpm1.12 + blebb | 1.793± 0.1492^a^ |
| Tpm4.2 + DMSO | 2.590± 0.2363 |
| Tpm4.2 + blebb | 1.658± 0.1334^b^ |
| Tpm3.1 + DMSO | 3.294± 0.4188 |
| Tpm3.1 + blebb | 2.228± 0.1961 ^c^ |
|  |  |

Cells were treated with DMSO or 50μM of blebbistatin for 30 min and the elastic modulus determined by indenting the cells in Peak Force Quantitative Nanomechanical Mapping (PFQNM) mode, according to the parameters described in Materials and Methods. 23-25 cells for each clone from *n*=3 independent experiments.

^a^Data shown represents the mean ± SEM with P<0.01 compared to Tpm1.12 + DMSO.

^b^Data shown represents the mean ± SEM with P<0.01 compared to Tpm4.2 + DMSO.

^c^Data shown represents the mean ± SEM with P<0.05 compared to Tpm3.1 + DMSO.
