# Supplementary material for: Cell Elasticity Is Regulated by the Tropomyosin Isoform Composition of the Actin Cytoskeleton
Source: PLoS One. 2015 May 15;10(5):e0126214. doi: 10.1371/journal.pone.0126214 (PMC4433179; doi:10.1371/journal.pone.0126214)
Supplement: S2 Table — Above Table is a summary of the data presented in Figs 1B , 2, 3 and 4. Highlighted in bold italics are the parameters found to be different relative to control cells. a P<0.05, b P<0.01, c P<0.001 and d P<0.0001 compared to control. (DOCX) [file pone.0126214.s009.docx]

S2 Table. Comparison of the elastic modulus with Tpm expression and F-actin quantitation.

| B35 clones | Elastic modulus  (kPa) | Tpm expression  (ng/μg total cell protein) | Phalloidin staining quantitation  (Total F-actin length per cell) | F/G actin ratio |
| --- | --- | --- | --- | --- |
| Control | 1.57 ± 0.15 | 3.96 ±0.16 | 916.2± 30.1 | 0.61 ± 0.08 |
| Tpm1.10 | 1.69 ± 0.21 | ***6.22±0.29^c^*** | ***677.4±48.24 ^d^*** | 0.31 ± 0.11 |
| Tpm1.11 | 1.98 ± 0.23 | 4.38±0.42 | ***597.9±22.64 ^d^*** | 0.39 ± 0.11 |
| Tpm1.12 | ***2.73 ± 0.29^c^*** | ***5.96±0.53 ^a^*** | 797.8±47.43 | 0.67 ± 0.11 |
| Tpm2.1 | 1.99 ± 0.15 | ***14.56±1.19^c^*** | 865.2±32.93 | 0.60 ± 0.01 |
| Tpm1.7 | 1.67 ± 0.17 | ***8.97±0.95 ^b^*** | ***496.5 ±47.57 ^d^*** | 0.83 ± 0.11 |
| Tpm4.2 | ***2.65 ± 0.21^d^*** | ***7.1±1.42 ^a^*** | 1049 ±35.08 | 0.53 ± 0.14 |
| Tpm3.1 | ***3.56 ± 0.26^d^*** | - 1. ***±0.42 ^d^*** | ***644.3 ±18.08 ^d^*** | ***1.01 ± 0.06 ^b^*** |
|  |  |  |  |  |

Above Table is a summary of the data presented in Fig. 1*B*, 2, 3 and 4. Highlighted in bold italics are the parameters found to be different relative to control cells. ^a^*P*<0.05, ^b^*P*<0.01, ^c^*P*<0.001 and ^d^*P*<0.0001 compared to control.
